# Targeted Treatment of Head and Neck (Pre)Cancer: Preclinical Target Identification and Development of Novel Therapeutic Applications

**DOI:** 10.3390/cancers13112774

**Published:** 2021-06-03

**Authors:** Anne M. van Harten, Ruud H. Brakenhoff

**Affiliations:** 1 Cancer Center Amsterdam, Otolaryngology-Head and Neck Surgery, Tumor Biology & Immunology Section, Vrije Universiteit Amsterdam, Amsterdam UMC, 1081 HV Amsterdam, The Netherlands; am.vanharten@amsterdamumc.nl or; 2 Sidney Kimmel Cancer Center, Department of Cancer Biology, Thomas Jefferson University, Philadelphia, PA 19107, USA

**Keywords:** HNSCC, targeted treatment, preclinical development

## Abstract

**Simple Summary:**

Head and neck squamous cell carcinomas (HNSCCs) develop from mucosal cells in the oral cavity, pharynx and larynx after either prolonged exposure to tobacco and alcohol, or a transforming infection with high-risk human papillomavirus (HPV). HPV-negative HNSCCs develop in a zone of premalignant mucosal cells centimeters in diameter and characterized by tumor-associated genetic changes, also referred to as ‘fields’, which can present as white leukoplakia lesions but are mostly invisible. Patients with HPV-negative HNSCC have an overall 5-years survival rate of 50–60%, despite application of intense treatment protocols, and current treatment regimens seem to have reached their plateau. Recently, immunotherapy using immune checkpoint inhibitors has been introduced, but seems effective in only some patients. Targeted treatments have failed to find their way into the clinic while novel therapies are urgently awaited that could target the tumor as well as the precancerous cells. However, recent data suggest that we are at the dawn of a new era. This review focusses on the preclinical identification of druggable targets for therapy for HPV-negative HNSCC and their preceding precancerous changes.

**Abstract:**

Head and neck squamous cell carcinomas (HNSCC) develop in the mucosal lining of the upper-aerodigestive tract. In carcinogen-induced HNSCC, tumors emerge from premalignant mucosal changes characterized by tumor-associated genetic alterations, also coined as ‘fields’ that are occasionally visible as leukoplakia or erythroplakia lesions but are mostly invisible. Consequently, HNSCC is generally diagnosed *de novo* at more advanced stages in about 70% of new diagnosis. Despite intense multimodality treatment protocols, the overall 5-years survival rate is 50–60% for patients with advanced stage of disease and seems to have reached a plateau. Of notable concern is the lack of further improvement in prognosis despite advances in treatment. This can be attributed to the late clinical presentation, failure of advanced HNSCC to respond to treatment, the deficit of effective targeted therapies to eradicate tumors and precancerous changes, and the lack of suitable markers for screening and personalized therapy. The molecular landscape of head and neck cancer has been elucidated in great detail, but the absence of oncogenic mutations hampers the identification of druggable targets for therapy to improve outcome of HNSCC. Currently, functional genomic approaches are being explored to identify potential therapeutic targets. Identification and validation of essential genes for both HNSCC and oral premalignancies, accompanied with biomarkers for therapy response, are being investigated. Attentive diagnosis and targeted therapy of the preceding oral premalignant (preHNSCC) changes may prevent the development of tumors. As classic oncogene addiction through activating mutations is not a realistic concept for treatment of HNSCC, synthetic lethality and collateral lethality need to be exploited, next to immune therapies. In recent studies it was shown that cell cycle regulation and DNA damage response pathways become significantly altered in HNSCC causing replication stress, which is an avenue that deserves further exploitation as an HNSCC vulnerability for treatment. The focus of this review is to summarize the current literature on the preclinical identification of potential druggable targets for therapy of (pre)HNSCC, emerging from the variety of gene knockdown and knockout strategies, and the testing of targeted inhibitors. We will conclude with a future perspective on targeted therapy of HNSCC and premalignant changes.

## 1. Introduction

Head and neck squamous cell carcinoma (HNSCC) ranks in the top ten of all cancers with respect to incidence, and encompasses the tumors that develop in the mucosal lining of the oral cavity, oropharynx, hypopharynx, and larynx. Annually 700,000 new cases are diagnosed worldwide. The incidence is gender biased; HNSCC occurs about two-times more often in males than in females [1]. This bias may be related to the major classical risk factors for HNSCC; carcinogen exposure from tobacco smoke and (excessive) alcohol consumption, with concomitant exposure increasing the risk [2,3,4]. A second risk factor for HNSCC is infection with a high-risk type human papillomavirus (hrHPV), particularly for tumors arising in the oropharynx [2,3,4,5,6,7,8]. Finally, patients with certain hereditary syndromes may predispose to HNSCC development, of which the most prominent example is the genetic instability syndrome Fanconi anemia (FA) [9,10,11,12,13]. 

The presence or absence of hrHPV analyzed by surrogate marker p16^Ink4A^-immunostaining, often followed by HPV DNA PCR, is currently used to classify tumors in the oropharynx in HPV-positive and HPV-negative disease [3,4]. These subgroups differ in progression-free and overall survival as well as in clinical and molecular characteristics [4,14,15,16]. The most recent data from a Dutch cohort of 1204 oropharyngeal squamous cell carcinoma (OPSCC) patients revealed a 5-years overall survival of 79% for the HPV-positive group and of 43% of the HPV-negative group [17]. Consequently, HPV-positive and HPV-negative OPSCC are considered as separate disease entities. The difference in prognosis has led furthermore to treatment de-escalation trials for HPV-positive HNSCC, although this has not yet resulted in a more personalized treatment based on HPV status [18,19]. 

As mentioned above, the major risk factors for HPV-negative HNSCC are tobacco and alcohol exposure [2,4,20,21]. Cancer risk relates to exposure, generally summarized as pack years and unit years, and particularly the combination of smoking with alcohol exposure increases risk synergistically [22]. However, HPV-negative tumors may also occur occasionally in patients who hardly smoked or consumed alcohol, and the reason for cancer development in these cases is still elusive [2,23]. Patients generally present with tumors *de novo*, but molecular research during the last decades revealed that HPV-negative HNSCC develops in premalignant changes in the mucosal lining of the head and neck region [4,24,25,26,27,28,29], which, however, are mostly not visible by eye. 

The premalignant cells cover large areas of the mucosal lining with dimensions of multiple centimeters in diameter, and contain some of the tumor-associated genetic changes (mutations and copy number aberrations) that are also observed in the majority of HNSCC [4,29,30,31]. These cells form a contiguous premalignant “field” that is mostly invisible to the naked eye but may be microscopically identified as dysplasia in surgical margins of excised tumor specimen. Fields can also be identified per definition using genetic markers on DNA obtained from surgical margins or cells obtained through oral brushing [29,30,31]. Only a smaller subgroup of premalignant fields are macroscopically visible as leukoplakia (white lesions) or erythroplakia (red lesions), which occur with a prevalence of 0.1–0.2% or 0.01–0.02%, respectively [4,32,33,34]. Sometimes multiple independent premalignant fields are found in a patient, harboring different gene mutations or genomic aberrations [4,24,26]. The fields can eventually evolve into a squamous cell carcinoma. As most of these fields are centimeters in diameter but not visible to the naked eye, fields may remain undetected and stay behind when tumors are excised. When premalignant fields stay behind, new tumors could form in these fields, which are clinically diagnosed as local recurrences when they occur within a distance of 2 cm from the original tumor and within a timespan of less than 3 years, or as a second primary tumor when located more than 2 cm from the original tumor or after 3 years [4,35]. Ideally, to prevent tumors developing in these fields, (targeted) treatments are needed to eradicate these cells before they transform, often again, into a malignancy. The treatment of premalignant fields is challenging as most are not macroscopically visible and their dimensions are covering large mucosal areas. Smaller visible abnormalities can be resected or treated with laser therapy, but often recur after treatment [33]. Visible or invisible, the curative treatment of premalignant fields remains problematic.

## 2. Current Treatment Protocols of HNSCC

### 2.1. Surgery and (Chemo-)Radiotherapy

During the last two decades, the therapeutic arsenal to treat cancer has been changing rapidly, and treatment of a variety of malignancies is increasingly based on tumor genetics, thereby aiming to employ personalized strategies with targeted agents, to reduce toxicity and enhance therapeutic efficacy [36,37]. Despite great efforts to uncover new targeted treatments that could find their way into the clinic, the mainstays of HNSCC treatment remain surgery and radiotherapy, the latter with or without concomitant cisplatin-based chemotherapy. Treatment planning is currently still based on site, tumor stage, imaging and post-operative histological findings, but not on genetics or hrHPV presence.

Albeit treatment protocols are generally intense and may cause disfigurements and toxicities in patients, responses remain somewhat disappointing [38]. Of all tumors, approximately 30% are diagnosed at an early stage [2,39], and these are usually treated with single modality treatment that comprise either surgical resection or radiotherapy, depending on the tumor site [40]. Complete cure is often obtained after treatment, and the 5-years survival rate is around 90% [41]. 

**Figure 1:**
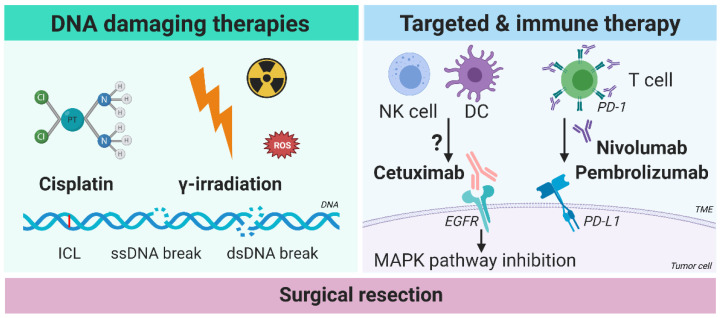
Approved and clinically applied treatment interventions for HNSCC. Early stage HNSCC is treated with either surgery or radiotherapy with photons or protons, while advanced stage HNSCC is treated by either upfront cisplatin-based chemoradiotherapy and salvage surgery when required, or upfront surgical resection with post-operative (chemo-)radiation. For patients unfit to receive cisplatin, the anti-EGFR monoclonal antibody cetuximab can be applied. Furthermore, patients with recurrent or metastatic disease are treated by the EXTREME protocol and with anti-PD-(L)1 antibodies, such as nivolumab and pembrolizumab, although response rates are low and clinical biomarkers for response are still under investigation. Abbreviations: ROS: reactive oxygen species, ICL: inter-strand crosslink, ssDNA break: single strand DNA break, dsDNA break: double strand DNA break, NK cell: natural killer cell, DC: dendritic cell, T cell: T lymphocyte, PD-1: programmed cell death protein 1, TME: tumor microenvironment, EGFR: epithelial growth factor receptor, PD-L1: programmed death-ligand 1, MAPK: mitogen-activated protein kinase.

However, the majority of patients (70%) present with advanced stages of disease, with regional lymph node metastasis or even metastases at distant sites [2,39]. These more advanced tumors are treated either with concomitant chemoradiotherapy and when required with surgical salvage, or with upfront surgery combined with post-operative (chemo)radiotherapy. In some centers neoadjuvant (chemo)radiotherapy followed by surgery may be applied [40,42]. Cisplatin has been the primary choice of chemotherapy since 1977 [43], and is combined with concomitant locoregional radiotherapy. For recurrent-metastatic disease and patients unfit for platinum-based therapy, immunotherapy with anti-PD-(L)1 antibodies, anti-EGFR targeting antibody cetuximab, or invasive multidrug chemotherapies are being applied (Figure 1).

Cisplatin is the mostly applied cytotoxic drug in chemotherapy regimen, which is often applied in combination with radiotherapy. The drug is able to form both intra-strand and inter-strand crosslinking bridges between the two complementary DNA strands of both the genomic and mitochondrial DNA [44,45]. This covalent inter-strand crosslink hampers DNA replication as the replication fork is challenged to pass the DNA crosslink. The key pathway to resolve such DNA crosslinks is the FA/BRCA-pathway (Figure 1) [11,46,47]. Cisplatin acts as radiation-sensitizer, but overall responses differ between tumors. A biomarker or biological explanation for response to cisplatin is unknown other than a defective FA/BRCA pathway [48]. Many HNSCC patients suffer from cisplatin-induced toxicity, and consequently they are frequently hospitalized and can often not sustain the full treatment protocol. Lastly, tumors are or may become resistant to cisplatin.

Most HNSCC patients receive irradiation with photons (IR) in approximately two Gray fractions and up to a total dose of 70 Gray [21,49,50,51]. Radiation (RT) induces the formation of DNA peroxides by water radiolysis in the presence of oxygen, generating reactive oxygen species (ROS) which induce a high number of single strand DNA (ssDNA) breaks (Figure 1) [52,53]. These breaks lead to the stalling of replication forks and G2/M-checkpoint activation. DNA damage is induced by both the stalled replication forks at ssDNA breaks and the formation of double stranded DNA (dsDNA) breaks during replication when ssDNA breaks that have not been repaired turn into dsDNA breaks [54,55]. Radiation-induced dsDNA damage is commonly repaired by non-homologous end joining (NHEJ) and microhomology-mediated end joining (MMEJ) [56,57,58,59,60]. NHEJ and MMEJ are error-prone DNA repair mechanisms, introducing mutations that are potentially lethal. Nonetheless, it has also been described that MMEJ may contribute to IR-resistance [58].

### 2.2. Bio-Radiotherapy

In 2007, the FDA approved chimeric monoclonal antibody cetuximab (Erbitux) for treatment of HNSCC (Figure 1) [61,62]. Cetuximab targets the membrane protein epithelial growth factor receptor (EGFR). Although the exact mechanism of action is not completely understood in head and neck cancer, it hinders the binding of EGF to the receptor and thereby inhibits downstream signaling pathways such as the Ras-MAPK-ERK pathway [61]. EGFR overexpression is frequently found in HNSCC, which is in some cases associated with amplification of chromosome 7p [4]. However, single agent response rates remain low in clinical trials (13%) and biomarkers that predict therapy outcome with cetuximab in HNSCC are unidentified [62,63]. It has also been suggested that cetuximab mainly acts as an activator of the immune system in HNSCC patients, thereby acting as a bridge between tumor cells expressing EGFR and immune cells such as CD16-positive NK-cells and dendritic cells (reviewed in [64,65]). The lack of biomarkers predicting response, together with the observation that EGFR small molecule inhibitors such as gefitinib are not particularly effective in the treatment of HNSCC, may point towards an immunological response rather than a molecular response to cetuximab treatment [66,67].

### 2.3. Immune Checkpoint Inhibitors

Evading the immune response is a commonly accepted hallmark of cancer [68], and HNSCC is known to be very immune suppressive [69]. By overexpression or downregulation of certain immune-associated membrane receptors, tumor cells become unrecognizable to the immune system [65,69,70,71]. One of these immune-suppressive systems is catalyzed by the interaction between PD-1 on the lymphocytes in the tumor microenvironment (TME) and its ligand PD-L1 on the tumor cells. The development of monoclonal antibodies, such as nivolumab (Opdivo^®^) and pembrolizumab (Keytruda^®^) directed against PD-1, have been shown to be an effective immunotherapeutic strategy in HNSCC [69] (Figure 1). These immune checkpoint inhibitors have been FDA approved for HNSCC since 2016 [72] for recurrent and metastatic disease, and will become more prominent in upfront treatment protocols. Tumor-specific PD-L1 expression is hypothesized to be a predictive biomarker for response, but results are inconclusive [73,74]. It is promising that the response rate in HNSCC to PD-1 antibody treatment is around 25% in both HPV-positive and HPV-negative HNSCC [75]. A recent study with a murine oral squamous cell carcinoma model using 4NQO exposure indicated that PD-1 antibody treatment inhibited progression of the premalignant lesions into carcinoma, which was to a lesser extent also observed in PD-L1 knockout mice treated with 4NQO [76]. This study suggests that PD-(L)1 antibody treatment could be effective in a preventive setting, although data from clinical trials will be important to test this hypothesis. It should be noted that antibody infusion is a rather invasive procedure for treating premalignant changes, as is the toxicity profile of these immune checkpoint inhibitors [77].

## 3. Molecular Landscape of HNSCC

### 3.1. Copy Number Alterations

The majority of HNSCC tumors are characterized by a high level of genomic instability, in part resulting from frequent inactivation of cell cycle control [78], which is reflected by many copy number alterations. In the vast majority of HNSCC, gains of chromosomal arms 3q, 5p and 7p, and losses of 3p, 4p and 18q are observed, irrespective of HPV status [3,79,80,81,82,83,84]. Focal amplifications of 3q26/28 are associated with over-expression of *TP63, SOX2* and *PIK3CA* [3,82,85] and lymph node metastasis is associated with the loss of 4p in oral cancers [86]. HPV-negative HNSCC often contains copy number (CN) gains of chromosomes 8q, 9q and 11p, and CN losses of 7q, 8p, 9p, 11q and 18p [3,79,80,81]. Some of the CN alterations in HPV-negative tumors are already present in premalignant cells [3,4,26,87]. Cell cultures obtained from premalignant fields, including leukoplakia and erythroplakia lesions, contain CN losses in 3p, 8p and 9p21, and amplifications of 3q and 8q, which are also often observed in HNSCC [3,26,87,88].

### 3.2. Cancer Driver Genes in HNSCC

Comprehensive genomic profiling of HNSCC by the TCGA consortium emphasized the large number of tumor suppressor genes that are inactivated by mutations or chromosomal aberrations, and these studies also highlighted the tremendous heterogeneity of HNSCC [3,38,89,90]. Driving oncogene mutations are largely underrepresented in HNSCC [3], hampering the development of targeted treatments that exploit the concept of ‘oncogene addiction’.

Loss of function of tumor suppressor genes *TP53* and *CDKN2A* (p16^Ink4A^) through gene mutation, methylation, or in the case of *CDKN2A* through focal loss of 9p21, are often found in both premalignant cells and HPV-negative HNSCC, and occur early in oncogenesis [3,26,29]. HPV-positive tumors typically lack mutations in *TP53* and *CDKN2A*, as the viral oncogenes E6 and E7 block the same signaling pathways. The fact that HPV-positive tumors have a favorable prognosis and are typically *TP53* wild type, interferes with the analysis of *TP53* mutations in relation to clinical outcome. Overall survival of HNSCC patients correlates poorly with *TP53* status particularly when stratified for HPV, conflicting with earlier assumptions when HPV status was not considered [3,78,91,92]. Both *CDKN2A* and *TP53,* through its downstream target p21^Cip1^, are important inhibitors of cyclin-CDK complexes and can cause cell cycle arrest. Loss of function of these genes thus results in diminished G1/S-checkpoint control and deregulated cellular proliferation, which is considered an important hallmark of cancer [68,93]. Besides *TP53* and *CDKN2A*, gene mutations in *FAT1, CASP8, AJUBA, PIK3CA, NOTCH1, KMT2D, NSD1, TGFBR2* and *HRAS* are observed in HPV-negative HNSCC [3]. Of note, alterations of *NOTCH1* in cancer can both be oncogenic or imply loss of function depending on the context, but *NOTCH1* mutations in HNSCC are generally considered as loss of function mutations and therefore *NOTCH1* acts as tumor suppressor [94]. *PIK3CA* mutations are found in both HPV-positive and -negative tumors, but mutational profiles differ. Amino acid substitutions E542K and E545K in the helical domain of *PIK3CA* are predominantly found in HPV-positive HNSCC, while *PIK3CA* mutations in HPV-negative HNSCC are generally found in the kinase domain [85,95,96].

Of the most frequently mutated genes in HNSCC, only those with oncogenic mutations in *PIK3CA* and *HRAS* can be targeted with small molecule inhibitors that are currently in preclinical testing for HNSCC and other malignancies (see Section 4). Unfortunately, only a minority of HPV-negative HNSCC harbor either a *PIK3CA* or *HRAS* mutation, and therefore only a small patient group might benefit from a therapy regimen targeting these mutations [3].

It stands out that most of the frequently mutated genes in HNSCC such as *TP53, CDKN2A, CCND1, HRAS, PIK3CA, PTEN* and *RB1* are responsible for increased cell proliferation and deregulation of cell cycle control [3]. In addition, mutations in *CASP8* as well as in *TP53, HRAS* and *PIK3CA* contribute to evasion of apoptotic cell death, all typical hallmarks of cancer [68]. Since HNSCC is mainly driven by mutations in tumor suppressor genes, these alterations in cell cycle progression and escape of cell death may serve as potential vulnerabilities to the efficient eradication of the tumor cells, as well as premalignant cells, with appropriately selected targeted agents.

## 4. Classic Approaches to Identifying Targets for Therapy

We define ‘targeted therapy’ as the therapeutic exploitation of a specific cellular vulnerability in the context of the somatic genetic changes in the tumor cells. Generally, three concepts are considered for the application of targeted therapies for different malignancies: oncogene addiction, synthetic lethality, and collateral lethality (Figure 1). We will discuss these principles and their feasibility in the preclinical development of targeted therapies for HNSCC.

### 4.1. Oncogene Addiction

**Figure 2:**
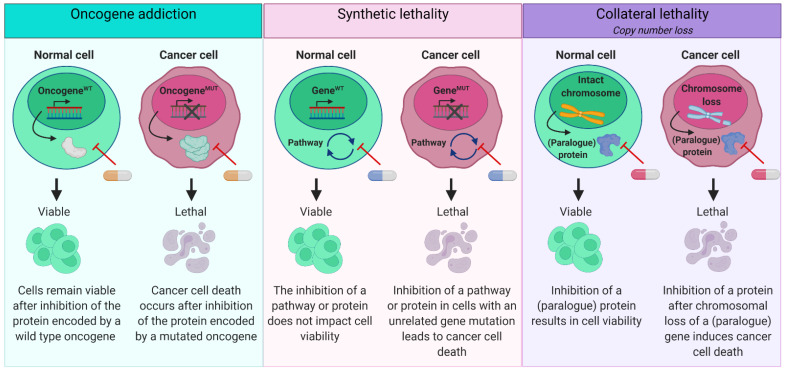
Concepts to identify therapeutic vulnerabilities in cancer cells. Left panel*:* Oncogene addiction. When a cancer cell harbors an activating mutation of an oncogene, a cancer cell may become completely dependent on this protein product, resulting in cell death upon targeted inhibition. Inhibition of the same albeit wild type protein impacts survival of normal cells less or not at all; these cells do not rely on this protein only for survival. Middle panel*:* Synthetic lethality. Inhibition of either two proteins or pathways with comparable function does not affect cell survival in normal cells. However, when a cancer cell harbors a gene mutation that inactivates a protein in such a pathway, cancer cells may become fully dependent on the other pathway. Inhibition of the remaining functional pathway will induce specific cancer cell death. Right panel*:* Collateral lethality. When a tumor suppressor gene is lost through loss of the chromosomal region, other neighboring genes located at that region may become dose-limiting and cause increased sensitivity for inhibiting drugs. Likewise, when a gene is lost or partially lost due to copy number losses, inhibition of a paralogue gene may specifically affect cancer cell viability. Abbreviations: WT: wild type, MUT: mutant.

Activating mutations of oncogenes may lead to the dependency of the cancer cell on this hyper-activation of the subsequent gene or the pathway it acts in [97]. Oncogene addiction forms an excellent vulnerability that can be targeted by specific inhibitors, and will lead to cancer-specific cell death, while untransformed cells may not be affected as they may rely on backup pathways (Figure 2, left panel) [98]. Targeting the *BRAF*^V600E^ mutation in melanoma using tyrosine kinase inhibitor vemurafenib dramatically changed the clinical treatment of melanoma [99]. Despite initial spectacular results, it also became apparent that resistance is often observed. Moreover, the *BRAF*^V600E^ and vemurafenib story has made crystal clear how critical it is that the pathways are understood in the smallest detail to overcome potential problems such as, in this case, the *BRAF*-paradox; inhibition of B-Raf with vemurafenib caused skin tumors in normal skin by inhibitor-induced refitting of the Raf pathway [100]. Despite these considerations, oncogene addition remains a promising concept in cancer therapy.

Unfortunately, the carcinogenesis of HNSCC is mainly driven by loss of tumor suppressor function [3], and therefore exploitation of oncogene addiction as a vulnerability concept is limited, but nonetheless explored. Oncogene *HRAS* is mutated (*HRAS^mut^*) in about 4% of HNSCC [3]. Two phase II clinical trials (NCT03719690, NCT02383927) are currently being conducted to investigate the efficacy of tipifarnib, a selective inhibitor of farnesyltransferase that was shown to be an effective strategy for *HRAS^mut^* HNSCC in a preclinical study [101]. A second oncogene, *PIK3CA*, is mutated in 20% of HNSCC, but only a minority of mutations are activating mutations [3,102]. PIK3CA acts in the PI3K-AKT-mTOR pathway that stimulates cell survival upon activation. Different mTOR inhibitors (everolimus and temsirolimus) and PI3K inhibitors (PX-866 and buparlisib) have been tested in phase II clinical trials, but response rates were disappointing and severe grade 3/4 side effects were observed (reviewed in [103]). A phase III clinical trial is currently being conducted (NCT04338399) in which PI3K inhibitor buparlisib is administered with paclitaxel in patients with recurrent or metastatic HNSCC.

The most obvious molecular candidate for a targeted approach in HNSCC seems to be EGFR. Of note, most HNSCCs show overexpression of EGFR, but never activating point mutations, which may explain the lack of response to EGFR-targeting kinase inhibitors such as gefitinib and erlotinib [4,66], suggesting that HNSCC cells might not be addicted to EGFR. Cetuximab, a human-mouse chimeric antibody directed against EGFR, has been registered for HNSCC, but it remains unclear whether its working mechanism relates to EGFR inhibition or activation of the immune system. Moreover, for HPV-positive disease, treatment with cetuximab is inferior to cisplatin and seems not to increase response rates [18,19].

### 4.2. Synthetic Lethality

As HNSCC is the main result of tumor suppressor gene inactivation, we have to rely on alternative concepts for targeted therapy approaches, such as synthetic lethality (Figure 2, middle panel). A classic example of synthetic lethality is the vulnerability to PARP inhibition in homologous recombination (HR) deficient tumors [104,105]. Neither the inhibition of PARP alone nor the loss of homology-directed repair impacts cell survival in normal cells. The loss of an HR gene such as *BRCA1* or *BRCA2* induces breast cancer oncogenesis. Additional loss of the second allele causes these tumors to be HR-deficient. When PARP function is inhibited by PARP inhibitors in these HR-deficient tumors, cell death is induced. In normal HR-proficient cells, HR-directed repair rescues PARP inhibition.

During the past decade, synthetic lethality has been exploited as a therapeutic approach for most malignancies including HNSCC, but so far with limited success. Although synergy is observed when combining two treatments (radiotherapy supplemented with cisplatin, Aurora inhibition combined with Wee1 inhibition, Haspin knockout combined with Aurora inhibitor [106,107,108], and many others (see Section 7), these do not follow the classical synthetic lethality concepts, as in these combination treatments one drug sensitizes the response to the other. In the case of classical chemoradiotherapy with cisplatin and irradiation, both induce DNA damage. Nonmalignant cells will respond by cell cycle arrest and DNA repair, but these controls are diminished in cancer cells due to genomic alterations, therefore cancer cells suffer from additional intrinsic DNA damage by replication stress from the uncontrolled entry into S-phase. These treatments are obviously to a lesser extent also toxic to nonmalignant cells with proper cell cycle control, creating a small therapeutic window, in the case of radiotherapy enhanced by image-guided planning and intensity modulation.

Classic synthetic lethal interactions have not yet led to clinical implementation as new therapies for HNSCC. However, exploiting rewired pathways such as those that regulate cell cycle control in cancer cells by targeting an additional cell cycle regulating protein also follows the concept of synthetic lethal interactions. Recent studies suggest that loss of cell cycle control by p53 and/or p16^Ink4A^ loss of functions sensitize (pre)HNSCC cells to Wee1, Chk1 and PLK1 inhibition [84,109,110,111,112]. Normal cells may stay arrested in G1/G0, while cancer cells are forced to enter S-phase and progress through the cell cycle and have to rely on the rewired control mechanisms that act during DNA replication and G2-M. When these rewired mechanisms are inhibited, the cancer cells die.

### 4.3. Collateral Lethality

Tumor suppressor genes become inactivated during carcinogenesis through mutations or genomic deletion. In case of genomic deletion, a larger part of the chromosome is generally deleted and neighboring genes may be lost in this process. The collateral loss of these passenger genes can be exploited for therapy since these genes may be involved in cellular processes, while the gene dosage and associated protein expression has now been halved in the cancer cells. Loss of such passenger genes may also be compensated through redundancy by paralogue genes as postulated by Muller et al. [113]. Subsequent interference with the paralogue gene may lead to decreased cell survival (Figure 2, Right panel). To identify redundant paralogue genes after loss of a passenger gene, integrated analysis is required to match genomic copy number losses with vulnerabilities to interference with known paralogues [110,113,114,115].

We previously reported on indications that collateral lethality may occur in premalignant oral cells and HNSCC tumor cells in a customized screen in multiple cell lines models with 319 siRNAs, preselected from prior HNSCC lethality screens, [110]. Although we were able to identify collateral lethality for some of these hits, no druggable targets were found in this small RNA interference screen. More comprehensive screening methods that integrate copy number alterations with functional data from high-throughput genetic screens need to be applied to pinpoint new therapeutic avenues based on collateral lethality to treat HNSCC.

## 5. Identification of Essential Genes in (Pre)HNSCC Cells by Descriptive and Functional Genomics

Fundamental understanding of the biology of the tumor cells is required to allow preclinical identification of vulnerabilities of HNSCC, and the selection of biomarkers for clinical stratification. Together these form the initial steps towards better and more personalized treatments for HNSCC. The descriptive genomics data established by consortia such as the TCGA [3], which summarize the driver mutations and genomic changes causing HNSCC in great detail, have revealed a wealth of new molecular information (see below). The TCGA aimed to identify new driver genes that could be targeted by small molecule inhibitors, but the identification of new therapeutic targets was insignificant due to the absence of oncogene drivers in HNSCC. However, new technological developments have allowed for a more functional genomics approach, and descriptive and functional genomics revealed complimentary insights into the drivers of HNSCC. To obtain unbiased data on essential genes and synergistic targets in tumor cells, (kinome) short hairpin RNA (shRNA) drop-out screens, microRNA expression screens, genome-wide array-based siRNA screens, CRISPR-Cas9 knockout screens and drug library screens are being used to identify essential genes in HNSCC cells and targeted inhibitors.

### 5.1. shRNA (Kinome) Drop-Out Library Screens

Small interference RNAs (siRNAs) are synthetic 20–25 nucleotide double-stranded RNA molecules that are complementary to gene transcripts, and cause degradation of the transcript or inhibition of translation upon binding [116]. Short hairpin RNAs (shRNAs) are the cloned versions of these siRNAs, inserted in plasmid or lentiviral vectors [117]. Both siRNAs and shRNAs can be pooled in so called libraries that target the whole genome or a part of the genome. Functional RNA interference kinome screens, are used to identify targetable kinases. Cells are plated in large culture dishes, infected with a lentiviral library containing the pooled shRNAs, and grown for a prolonged time after resistance-marker selection. DNA sequencing reveals the relative increase or decrease of the shRNA constructs that have a stimulating or inhibiting effect on proliferation, between freshly infected cells (t0) and study endpoint. Further validation of the identified hits is required to ensure validity and essentiality, since either off-target effects are observed with shRNAs that result in false-positive hits, or cells had been infected with multiple shRNAs leading to a specific lethality [118]. The incomplete knockdown of the target genes by shRNAs (and siRNAs) mimics drug inhibition best, but may cause essential genes to be missed in these screens. Despite these considerations, it stands out that in the shRNA screens conducted in HNSCC, many genes regulating the cell cycle, as well as DNA damage response, emerged as essential [119,120,121,122,123].

### 5.2. Genome-Wide Array-Based siRNA Screens and microRNA Expression Library

Pooled shRNA screens are relatively time-efficient compared to array-based siRNA screens that demand large scale experiments with extensive robotics (see below), but amplification of the shRNA libraries may cause the library not to be fully representative. In addition, shRNAs are processed as microRNA genes, and consequently may behave as miRNAs, the small 20–25 base pairs of single stranded RNAs that are naturally expressed in cells and regulate gene expression by targeting multiple RNA transcripts [116,124]. An alternative is array-based siRNA screens with synthetic and optimized RNA molecules, which are not processed but directly act on the transcripts in the cells. These screens are conducted in 96 or 384 well plates and only one siRNA pool (a mix of a few siRNAs complementary to the same transcript) targeting only one single gene is administered per well, together with a pre-optimized lipid transfection reagent. Cells are added and usually 96 h after transfection cell viability can be measured and essential hits can be identified by bioinformatic analysis [125]. Several genome-wide and sub-genome custom library siRNA screens have been conducted in HNSCC cell lines as well as premalignant oral cell lines to identify essential genes [109,110,126,127,128]. Again, many genes involved in cell cycle regulation, DNA damage response and mitotic spindle regulation have been identified as potential therapeutic candidates for HNSCC. In addition, siRNA screens have been conducted to identify biomarkers for therapy response or combination therapies [129]. To identify the hits that are tumor cell-specific, the siRNAs are also tested in nonmalignant cells. These unbiased screens also reveal tumor-specific hits that are not easily explained, such as splice factors or ribosomal genes [110,127]. Some genes appear to be essential for all cells including normal cells and are generally involved in protein homeostasis, such as ubiquitin genes, or protein trafficking. However, most genes target tumor cells more effective than normal cells.

A similar approach to array-based siRNA screens is the use of array-based microRNA expression libraries to identify putative targets for therapy. The overexpression of the microRNAs inhibits the expression of a variety of target genes, causing an effect on cell proliferation or other cellular processes. Expression of microRNAs miR-181a, −326, and −345 have been reported to specifically kill HNSCC tumor cells by decreasing ATM expression [130].

### 5.3. CRISPR-Cas9 Knockout Screens

The CRISPR-Cas9 genome editing approaches have accelerated functional genomic screens both to identify cancer cell vulnerabilities and elucidate gene function. CRISPR-Cas9 knockout screens, contrary to siRNA and shRNA libraries, enable complete knockout of target genes, which have become a game changer in the field of functional genomics. The Cas9 endonuclease is directed to specific loci in the genome (the PAM sequence) by so called guide RNAs (gRNAs) that have a sequence complementary to the gene of interest. The DNA break induced by Cas9 and the gRNA in the gene of interest is repaired by error-prone NHEJ, which leads to a deletion or insertion and functional knockout of the gene. Screens can either be using a pooled lentiviral library with cloned gRNAs, followed by library sequencing to identify depleted or enriched gRNAs targeting essential genes, or by using an array-based synthetic gRNA approach with viability readout [131]. Several reports have been published that utilized a CRISPR screen approach to uncover biological mechanisms and essential genes for therapy [106,132,133,134,135]. Furthermore, as part of the Cancer Dependency Map Project (the Wellcome Sanger Institute and the Broad Institute), several HNSCC cell lines have already been screened and these datasets are publicly available [136,137,138]. Similarly, to shRNA and siRNA screens, lethal hits can be missed in CRISPR screens, because of unspecific gRNAs or gRNAs that induce functional splice variants in the gene. The latest versions of libraries have been optimized, however, and are very specific. Limitations are that a fully active NHEJ system must be available in cells.

### 5.4. Drug Library Screens

Many investigators choose a shortcut and directly screen for cancer cell vulnerabilities by using drug libraries. Many commercial drug libraries are available to screen for targeted inhibitors that may impact survival of cell lines *in vitro*. Multiple array-based drug screens have been performed in HNSCC cell lines and primary HNSCC cells in 2D tissue culture, to identify synergistic drug combinations or new targets for therapy [139,140,141,142,143,144]. These screens prominently identified many cell cycle and DNA damage response inhibitors impacting survival of HNSCC cells. Vulnerability data from drug screens in many cancer cell lines including HNSCC are collected in datasets such as Genomics of Drug Sensitivity in Cancer (GDSC) (The Cancer Genome Project by the Wellcome Sanger Institute and Massachusetts General Hospital Cancer Center) [145]. It would be of interest to obtain survival data of premalignant oral cells with drug libraries to exploit treatments for high-risk premalignant changes. In addition, further research is needed to move these in vitro observations into clinical trials. The obvious limitations of drugs screens are that these are biased, in that many inhibitors have off-target effects and inhibit a multitude of proteins, which complicates the understanding of the workings mechanism of a particular drug and hampers insights into the underlying biological process.

### 5.5. Descriptive Genomics Technologies

Descriptive genomics encompasses large scale DNA analyses, transcriptomics and proteomics, and can be applied on both cultured cells and patient samples. These approaches describe and analyze the molecular landscape of cancer including somatic mutations and copy number alterations, profiles of methylation, gene expression, microRNAs, and proteins, the latter including post-translational modifications such as phosphorylation. Data are collected in publicly available databases, which allows for computational biology approaches by any research to uncover new tumor vulnerabilities and biomarkers for therapy. Several initiatives by large consortia to perform in depth profiling of a multitude of cancers have been initiated in the last decade, and both large scale genomics data and data on sensitivity to drug libraries are collected, by, for example, The Cancer Genome Atlas Network [3], The Human Protein Atlas [146] and L1000 [147] amongst others (reviewed in [148]). Initiatives must be large scale as the significance of modeled predictions on tumor classification or outcome directly increases with the number of analyzed samples. However, technical aspects such as sequencing depth and data of normal control samples also support the interpretation of these data. Several in silico analysis of HNSCC have now been published [142,149,150,151]. Furthermore, machine learning models are developed to predict HNSCC tumor progression [152], along with other HNSCC data sources as summarized by Willems et al. [153]. Predicted tumor vulnerabilities and potential biomarkers from computational approaches are however hypothesis-generating and these hypotheses will need to be tested by both in vitro and in vivo preclinical studies before translation into the clinic. To further utilize the abundance of data obtained through genomics, transcriptomics, and proteomics, together with tumor cell vulnerability data obtained through functional genomics approaches such as siRNA, CRISPR or drug library screens, computational platforms have been developed to identify disease- and subgroup-specific putative targets while estimating the efficiency and toxicity based on modeled predictions through in silico analysis [148]. Although the data is out there, understanding the underlying biological principles and translating these to new therapeutic avenues is a challenge for the future.

## 6. Changes in Cell Cycle in HNSCC

**Figure 3:**
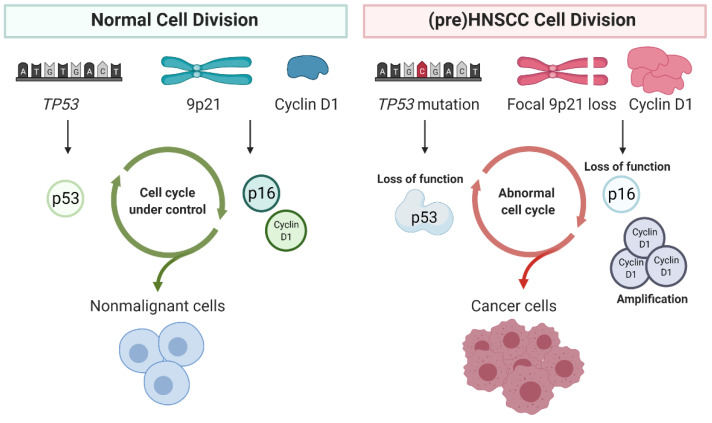
Cell cycle control in premalignant mucosal cells and HPV-negative HNSCC is impaired through frequent inactivation of p53 by (point) mutations. P16^Ink4A^ encoded by the *CDKN2A* gene at chromosome 9p21 is frequently inactivated by either mutations or promotor methylation, or focal homozygous losses of chromosome 9p21. Combined with the amplification of the *CCND1* gene which encodes for cell cycle regulating protein cyclin D1, these alterations result in loss of normal cell cycle control and increase tolerance for DNA damage and aneuploidy as often observed in cancer cells.

Unbiased screening for vulnerabilities in HNSCC cells has uncovered that inhibition of proteins that regulate cell cycle and DNA damage response impact survival of both premalignant oral cells and HNSCC (see above). A recent proteogenomic study using 108 HPV-negative HNSCC patient tumors strengthened this observation by uncovering that important drivers of HNSCC carcinogenesis act in the cell cycle [151]. The cell cycle is compromised in most HPV-negative HNSCC and premalignant cells through loss of function of p53, p16^Ink4A^ (mutations, methylations and focal losses of chromosomal locus 9p21) and frequent amplification of cyclin D1 (Figure 3) [3,26]. In normal cells, cellular growth stimulatory signals (mitogens) induce cyclin D1 expression and consequently CDK4/6 activation, Rb phosphorylation, E2F release and transition from G1- to S-phase. This is counteracted by p16^Ink4A^ induced arrest [154,155,156]. Cellular stress leads to increased stabilization of p53 through posttranslational modifications of both p53 and its E3 ubiquitin ligase MDM2 which becomes inactive by the modification. If DNA damage occurs, the ATM-Chk2 signaling cascade is activated [155,157,158,159,160,161,162,163,164,165,166,167]. The p53 protein acts as tetrameric stress-induced transcription factor and induces p21^Cip1^ expression, causing the inhibition of the cyclin-CDK complexes, and cells stay in G1-phase or arrest in S-phase and particularly G2-phase to support efficient DNA repair or induce apoptosis [160,161,163,167,168,169]. Unscheduled S-phase entry induces replication stress and subsequent DNA damage, and consequently cell death by p53 induction. Remarkably, knockout of p53 or p21^Cip1^ reduces the replication stress induced DNA damage, a counterintuitive finding in mouse cells after knockout of all Rb proteins [170]. This observation was made after starvation induced replication stress in engineered cells, which might differ from the real situation in cancer cells. Although the precise mechanisms remain unclear, most tumor cells as well as premalignant cells suffer from replication stress induced DNA damage.

Release from cellular stress inducers results in degradation of p53 by MDM2, and allows restart of cell cycle progression by multiple mechanisms [160,168]. Upon loss of p53, p16^Ink4A^ or both in cancer cells, the progression from G1- to S-phase is especially impacted due to loss of the G1-checkpoint. In addition, DNA damage induced cell cycle arrest is inhibited by the loss of p53 and, together with an altered expression of cyclin D1, HNSCC cells are unperturbed continuing from G1- into S-phase [3,4,171]. The involvement of cyclin D1 goes beyond complexing with CDK4/6 alone, since it is also involved in regulation of the DNA damage response through BRCA2, RAD51 and p21^Cip1^, and different chromatin modification pathways (as reviewed in [172]). Together, these frequently occurring genetic alterations result in a rewired cell cycle and deregulated DNA damage response, explaining the tumor vulnerability to regulators of these processes.

Many classical cytotoxic chemotherapeutic agents used in cancer therapy exploit alterations in cell cycle regulation in cancer cells. As mentioned above (Figure 1), the crosslinking agent cisplatin remains the first choice of chemotherapy in HNSCC combined with radiotherapy. As reviewed by Williams and Stoeber, cisplatin treatment efficiently affects DNA replication in S-phase and the subsequent G2-phase [173]. Cells arrest in the S- and G2-phase to allow time for DNA repair. Additionally, classically applied chemotherapeutic agents in standard oncological treatment protocols like 5-fluorouracil (5-FU, a thymidylate synthase inhibitor), methotrexate (an inhibitor of dihydrofolate reductase), irinotecan/campthotecin (inhibitors of topomerase I) and the RNR-complex inhibitor and nucleotide analogue gemcitabine, all affect DNA replication and S-phase progression [173,174]. Tubulin-targeting agents docetaxel, paclitaxel and vincristine furthermore interfere with mitotic progression by stabilizing the spindles [175]. Although these chemotherapeutics target both malignant and rapid proliferating untransformed cells and may cause severe toxicities, these therapeutics illustrate that the mechanisms underlying DNA replication and cell cycle progression harbor targetable vulnerabilities in HNSCC and other cancer cells (Table 1).

## 7. Clinical Perspective

### 7.1. Targeting the Cell Cycle for Therapy

During the last decade, a novel interest emerged in targeting the cell cycle in HNSCC (Table 1) as well as other cancers [155,176,177,178,179,180,181,182,183,184,185,186]. The therapeutic effects of classical cytotoxic agents and γ-irradiation already point to the efficacy of targeting the rewired cell cycle in cancer cells. To execute more effective targeted treatments protocols for HNSCC in clinical care, the research objective is to make treatments more efficient and with less collateral damage in nonmalignant cells. The application of functional genomic approaches by mRNA interference with siRNAs and shRNAs, microRNA expression libraries, high-throughput CRISPR-Cas9 screens, and drug library screens enabled the identification of essential genes specifically to HNSCC cells but at a lesser or no extent to nonmalignant cells [121,127,137,187,188]. Several S-phase and DNA damage related genes have been identified as potential targets for treatment of HNSCC as well as high-risk premalignant changes (Table 1). *ATM* mRNA knockdown was reported to be lethal when using microRNA expressing oligonucleotides [187]. The complexity of the ATM protein as well as redundancy in the kinase domain of ATM with other PIKK-family members, complicates the development of specific inhibitors with sufficient bioavailability *in vivo*. Nevertheless, a phase I clinical trial with the new ATM inhibitor AZD0156 is now conducted in solid tumors (NCT02588105) [111,187,189,190,191,192]. Similarly, PIKK-family member *ATR* was identified as an essential gene in HNSCC, but small molecule kinase inhibitors has lacked specificity in clinical trials so far. Recent developments uncovered highly potent small molecules against ATR and DNA-PK, and clinical trials are conducted in HNSCC with these inhibitors both as single agent and in combination (Table 1; ATR: NCT04576091, NCT04491942; DNA-PK: NCT04533750, NCT01353625) [48,111,177,193,194].

Interference of Aurora proteins, *FOXM1*, *KIF11* and *PLK1* showed promising results in vitro and in vivo in HNSCC, but these molecular targets are currently not tested in HNSCC in clinical trials [107,109,127,188,195,196,197,198,199,200,201,202,203,204]. Furthermore, therapeutic inhibition of S-phase regulator CDC7, the function of which is essential for origin firing and replication fork formation [205], is currently clinically tested in a phase I trial with CDC7 inhibitor LY3143921 in HPV-negative HNSCC patients (NCT03096054). Although the clinical application of monotherapy with CDK4/6 inhibitors might not be suitable for HNSCC, clinical trials in combination with cetuximab, radiotherapy, PI3K/mTOR inhibition or anti-PD-(L)1 antibodies are currently being conducted (NCT03065062, NCT03024489, NCT04000529) [206,207,208].

**Table 1:** Preclinical studies referring to druggable hits in cell cycle control.

Target	Inhibitor or Interference Method		HPV	Ref
ATM	Antisense oligodeoxynucleotides	*In vitro*	U	[189]
	Nanoparticles with AS-ODNs	*In vitro*	U	[190]
	Antisense oligodeoxynucleotides	*In vitro, in vivo*	Positive	[191]
	microRNA expression	*In vitro*	Negative	[187]
	AZD0156	*In vitro*	Negative	[209]
	KU-55933 +/− photons +/− protons	*In vitro*	Both	[210]
ATR	siRNA interference	*In vitro*	U	[193]
	AZD6738 +/− KU-0060648	*In vitro*	U	[48]
	AZD6738 +/− Paclitaxel or Cisplatin	*In vitro, in vivo*	Both	[194]
	AZD6738; VX-970	*In vitro*	Negative	[209]
	VE-821 +/− photons +/− protons	*In vitro*	Both	[210]
AURORA	siRNA interference +/− Paclitaxel	*In vitro*	Negative	[199]
	R763, Alisertib ^a^	*In vitro*	U	[200]
	Alisertib ^a^ +/− MG132	*In vitro, in vivo*	Positive	[201]
	Danusertib ^b^	*In vitro, in vivo*	Both	[188]
	Alisertib ^a^ +/− Adavosertib ^c^	*In vitro, in vivo*	Negative	[107]
	Alisertib ^a^; Danusertib ^i^	*In vitro*	Negative	[209]
	VX-680 +/− Haspin^KO^ or CHR-3464	*In vitro*	Negative	[106]
CDC7	XL413 +/− Cisplatin and Fluorouracil	*In vitro, in vivo*	Both	[202]
CDK4/6	Palbociclib + Cetuximab	Phase I trial	Both	[206]
	Abemaciclib +/− Torin2 or Everolimus	*In vitro, in vivo*	U	[207]
	Ribociclib +/− RT	*In vitro*	U	[208]
	Palbociclib + Cetuximab	Phase II trial	Negative	[211]
	Palbociclib + Cisplatin	*Phase I trial, in vivo, in vitro*	Negative	[212]
	Abemaciclib + Metformin	*In vivo*	U	[213]
	Palbociclib + Navitoclax	*In vitro*	Both	[214]
	Palbociclib	*In vitro*	Negative	[209]
Chk1/2	PF-00477736 +/− RT	*In vitro*	Positive	[215]
	AZD7762 +/− Cisplatin	*In vitro*	Negative	[216]
	siRNA interference	*In vitro*	U	[193]
	MK-8776 +/− Adavosertib ^c^	*In vitro*	U	[217]
	CCT244747 +/− RT +/− Paclitaxel	*In vitro, in vivo*	Both	[218]
	Prexasertib ^d^	Phase I trial	U	[219]
	AZD7762 or Rabusertib ^e^ or MK8776 + / − RT	*In vitro*	Positive	[220]
	Prexasertib ^d^ +/− RT +/− Cetuximab	*In vitro, in vivo*	Both	[221]
	AZD7762	*In vitro, in vivo*	Both	[198]
	Prexasertib ^d^	Phase I trial	Both	[222]
	siRNA interference; Prexasertib ^d^	*In vitro*	Both	[112]
	siRNA interference	*In vitro*	Negative	[126]
	siRNA interference; Rabusertib ^e^; Prexasertib ^d^; MK-8776; PF-477736	*In vitro*	Both	[111]
	PF-00477736 +/− Alpelisib ^h^	*In vitro, in vivo*	U	[223]
	shRNA; MK-8776 +/− Niraparib + / − RT	*In vitro, in vivo*	Both	[224]
	Prexasertib ^d^ +/− Cisplatin + / − RT	*In vitro, in vivo*	Both	[225]
	Prexasertib ^d^; MK8776	*In vitro*	Negative	[209]
	Prexasertib ^d^	*In vivo*	Both	[226]
DNA-PK	KU-0060648 +/− AZD6738	*In vitro*	U	[48]
	CC-115	*In vitro*	Negative	[209]
	KU-57788 or IC87361 +/− Olaparib or Veliparib +/− RT	*In vitro*	Both	[227]
	NU7441 +/− Olaparib	*In vitro, in vivo*	Negative	[228]
	KU-57788 +/− photons +/− protons	*In vitro*	Both	[210]
FOXM1	siRNA interference	*In vitro*	Positive	[203]
	Thiostrepton	*In vitro, In vivo*	Positive	[204]
KIF11	Ispinesib	Phase II trial	U	[195]
	siRNA interference; Ispinesib	*In vitro, in vivo*	Negative	[127]
PLK1	siRNA inteference +/− RT	*In vitro, in vivo*	Negative	[196]
	BI2536	*In vitro*	U	[197]
	Volasertib ^f^	*In vitro, in vivo*	Both	[198]
	siRNA interference; Volasertib ^f^; GSK461364; Rigosertib ^g^; HMN-214	*In vitro, in vivo*	Neg, preHN	[109]
	BI2536	*In vitro*	Negative	[209]
Wee1	Adavosertib ^c^	*In vitro, in vivo*	Both	[121]
	Adavosertib ^c^ +/− Cisplatin	*In vitro, in vivo*	Negative	[229]
	Adavosertib ^c^ +/− Cisplatin	*In vitro, in vivo*	Positive	[230]
	Adavosertib ^c^ +/− RT	*In vitro*	Positive	[220]
	Adavosertib ^c^ +/− Vorinostat	*In vitro, in vivo*	Negative	[231]
	Adavosertib ^c^	*In vitro*	Negative	[232]
	Adavosertib ^c^	*In vitro, in vivo*	Both	[198]
	Adavosertib ^c^	*In vitro, in vivo*	U	[233]
	Adavosertib ^c^ + Cisplatin + Docetaxel	Phase I trial	Both	[234]
	siRNA interference	*In vitro*	Negative	[126]
	Adavosertib ^c^	*In vitro*	Negative	[209]
	Adavosertib ^c^	*In vitro*	Positive	[235]
	Adavosertib ^c^ +/− Alisertib ^a^	*In vitro, in vivo*	Negative	[107]
	siRNA interference; Adavosertib ^e^	*In vitro*	Neg, preHN	[110]
	Adavosertib ^c^	*In vitro*	Positive	[236]
	Adavosertib ^c^ + Cisplatin	Phase I trial	U	[237]
	shRNA; Adavosertib ^e^ +/− Niraparib +/− RT	*In vitro, in vivo*	Both	[224]
*Ledgends*	*^a^ MLN8237*	*^f^ BI6727*	*RT radiotherapy*	
	*^b^ PHA-739358*	*^g^ ON-01910*	*U unknown*	
	*^c^ AZD1775/MK-1775*	*^h^ BYL-71*	*preHN premalignant oral cells*	
	*^d^ LY2606368*	*^i^ AMG900*	*KO CRISPR-mediated knockout*	
	*^e^ LY2603618*			

In several reports the susceptibility of HNSCC to Chk1 and Wee1 inhibitors has been published (Table 1) [107,110,111,112,121,126,193,198,209,215,216,217,218,219,220,221,222,223,224,225,226,229,230,231,232,233,234,235,236,237]. Chk1 is mainly involved in S-phase regulation upon stalled replication forks, which makes it a feasible target for cancers with high replication stress such as HNSCC [180,238,239,240,241,242]. Clinical trials with the Chk1 inhibitor SRA737 and the dual Chk1/Chk2 inhibitor prexasertib have been completed for treatment of solid tumors amongst which is HNSCC (NCT01115790, NCT02555644, NCT02797964).

Wee1 is a critical regulator of the G2/M-checkpoint through CDK1 phosphorylation, which inactivates the protein, but also plays a role in S-phase regulation by inactivating CDK2 phosphorylation against a background of replication problems [180,186,241,242]. The Wee1 inhibitor adavosertib is the only compound tested in clinical trials, including several studies in HNSCC, either as monotherapy or in combination with conventional chemotherapy and radiation (NCT04460937, NCT03028766) [177]. A phase I trial with promising results in HNSCC has been published [234].

Premalignant cells have been shown to be specifically vulnerable to PLK1 and Wee1 inhibition, whilst normal oral keratinocyte and fibroblast cells are not affected [109,110]. This strengthens the hypothesis that loss of p53, p16^Ink4A^ and to a lesser extent cyclin D1 amplification as seen in premalignant cells [26], sensitizes cells to these inhibitors, which may support the initiation of clinical trials. Particularly the application as monotherapy for treatment of high-risk premalignant changes characterized by morphological and genetic changes, might contribute to eradication of visible changes such as leukoplakias, but may also lead to less recurrent disease after treatment of the index tumor and improve overall survival rates of HNSCC patients.

Altogether, these preclinical and clinical trial data indicate the feasibility of targeting DNA replication and cell cycle progression in HNSCC. It should be noted, however, that the data in cell lines also point towards tumor-specific differences in response, possibly reflecting inter-tumor heterogeneity of HNSCC [90]. Clinical trials will provide important additional information on efficacy and biomarkers for patient selection in the future, as well as useful combination therapies. For many targets, small molecule inhibitors that show better bio-distribution and target-specificity in vivo and alternative therapeutic molecules such as protein degrading (PROTAC) molecules [243] are needed for more successful clinical implementation and increased responses.

### 7.2. Combining PD-L1 Antibodies with DNA Damaging Agents

Preclinical research has shown that activation of the cGAS/STING pathway after DNA damage enhances immune cell infiltration into the tumor microenvironment (TME) through secretion of pro-inflammatory type I interferon (IFN) and induction of a senescence-associated secretory phenotype (SASP) [244]. More data has been published recently indicating that degree of successful cGAS/STING pathway activation after classical DNA-damaging chemotherapeutics and radiation therapy influences treatment outcome through antitumor immunity (as reviewed in [245]). By inducing DNA damage and activation of T-cells, two hallmarks of cancer are being exploited, with potentially better responses and less therapy resistance [68,246]. To reduce toxicity as observed with classical chemotherapies and to enhance tumor-specificity, further research should determine whether combination of immune checkpoint inhibitors such as anti-PD-(L)1 antibodies with targeted agents improves HNSCC response rates and survival. As shown in other tumor types (reviewed in [246]), inhibitors targeting molecules such as ATR, Wee1 and Chk1, which induce replication-associated DNA damage and potentially cGAS/STING pathway activation through subsequent cytosolic DNA fragments in HNSCC, are promising candidates for combination treatments in HNSCC. Clinical trials are currently conducted combining ATR and Chk1 inhibitors with anti-PD-(L)1 antibodies in solid tumors including HNSCC (NCT04266912, NCT02264678, NCT04095273, NCT03495323) [246].

## 8. Conclusions

Survival rates for late stage HNSCC remain disappointing and while protocols for first-line treatment have been optimized in recent decades, this has not fundamentally improved survival since the implementation of cisplatin in 1977. The FDA approval of cetuximab in 2007 and two anti-PD-1 antibodies in 2016 have increased the arsenal, but the response rates still leave much to be desired and the lack of reliable biomarkers for response hamper implementation given the costs and associated toxicities of these new agents. Treatments with targeted agents have hardly been introduced in routine care as activating mutations in oncogenes are scarce in HNSCC, and clinical results so far were disappointing. In the last decade, through unbiased screening of siRNA and shRNA libraries, inhibitor libraries and more recently guide RNA libraries utilizing CRISPR-Cas9 technology, a better understanding of HNSCC vulnerabilities was established. Especially deregulation of the cell cycle in HNSCC has emerged as a candidate to be exploited for clinical use, not only to treat invasive cancers but also high-risk premalignant mucosal cells. In addition, combination therapies with immune checkpoint inhibitors and targeted agents that affect DNA damaging, such as ATR, Chk1 and Wee1 inhibitors, have potential to further increase response rates and survival. Better understanding of genomic alterations and loss of passenger genes will furthermore contribute to the identification of targets through synthetic and collateral lethality, which will expand our pre-clinical toolbox to uncover new HNSCC vulnerabilities in specific genetic backgrounds. Lastly, research should increase the focus on premalignant mucosal cells, and test vulnerability of these cells to new therapeutic compounds. By targeting these lesions together with the index tumor during treatment, recurrent and secondary tumors may be prevented. Furthermore, diagnosis and treatment of high-risk premalignant lesions by targeted inhibitors with their generally mild toxicity profiles could prevent the malignant progression of these precancers into difficult to treat HNSCC tumors.
